# Targeting ASCT2-mediated glutamine metabolism inhibits proliferation and promotes apoptosis of pancreatic cancer cells

**DOI:** 10.1042/BSR20212171

**Published:** 2022-03-16

**Authors:** Wenbin Wang, Haihua Pan, Feihua Ren, Hongxia Chen, Ping Ren

**Affiliations:** 1 Wuhan Sixth Hospital Affiliated to Jianghan University, Wuhan, Hubei, China; 2 School of Pharmacy, Hubei University of Science and Technology, Xianning, Hubei, China; 3 School of Public Health, Guizhou Medical University, Guiyang, Guizhou, China; 4 College of Medicine and Health Science, Wuhan Polytechnic University, Wuhan, Hubei, China

**Keywords:** apoptosis, ASCT2, glutamine metabolism, pancreatic cancer

## Abstract

Some tumor cells have a high rate of glutamine uptake and exhibit glutamine addiction. Alanine-serine cysteine-preferring transporter 2 (ASCT2) is a major mediator of glutamine supply in many tumor cells, but the underlying effects and mechanisms of ASCT2 in pancreatic cancer (PC) are largely unknown. Our results show that ASCT2 expression is significantly higher in PC than in normal pancreatic duct cells and pancreas. Utilizing the Kaplan–Meier Plotter database, a high expression of *SLC1A5* mRNA was significantly associated with poor overall survival (OS) in patients with PC. shRNA-mediated inhibition of ASCT2 function *in vitro* can significantly decrease glutamine consumption, α-ketoglutarate (α-KG) production and ATP generation and increase the reactive oxygen species (ROS) level. Moreover, the antioxidant N-acetylcysteine partially attenuated the increase in the ROS levels and reduced ATP generation. These data suggest that ASCT2 mediates glutamine metabolism and maintains redox homeostasis in PC. To further investigate whether ASCT2 is involved in PC cell growth, we blocked ASCT2 activity with the ASCT2 inhibitor l-γ-glutamyl-*p*-nitroanilide (GPNA) and silenced the expression of ASCT2 with specific shRNAs. We found that the growth of PC cells was significantly inhibited. Additionally, knockdown of ASCT2 induced apoptosis through the Akt/mTOR signaling pathway. Furthermore, the loss of ASCT2 in BxPC-3 cell xenografts significantly inhibited tumor growth *in vivo*, and this effect was associated with an increase in cleaved caspase-3 expression and a decrease in Ki67 staining. Taken together, our results show that ASCT2 may be utilized as a putative therapeutic target for PC.

## Introduction

Pancreatic cancer (PC) is a common gastrointestinal malignancy, and approximately 90% of PC originates from the ductal epithelium in the exocrine part of the gland [1]. It has become the seventh leading cause of cancer deaths worldwide [2], with 5-year survival hovering at approximately 5–10% and life expectancy at diagnosis of less than 5 months [3]. Surgery is effective in the treatment of early PC; however, only a very small proportion of PC patients (approximately 9–15%) are eligible for surgery [4]. Therefore, patients with advanced PC are limited to chemotherapy [5]. To date, gemcitabine treatment of PC is still the drug of choice, but a large number of patients do not respond to gemcitabine due to intrinsic or acquired chemoresistance [6]. Thus, it is necessary to explore the potential drivers of this cancer in order to discover new therapeutic targets for treating PC.

Abnormal glutamine metabolism is one of the hallmarks of cancer cells. Cancer cells rely on increased glutamine uptake, which provides an unlimited growth advantage [7]. Alanine-serine-cysteine transporter 2 (ASCT2; also known as SLC1A5), which belongs to the amino acid transport system ASC family, is the main glutamine transporter which determines the levels of intracellular glutamine [8]. Meanwhile, numerous studies have shown that compared with normal controls, a variety of tumor cells, such as liver cancer, lung cancer, melanoma, colorectal cancer and prostate cancer, exhibit significantly increased expression of ASCT2 [9–13]. These studies also showed that antisense RNA interferes with ASCT2 expression and significantly inhibits the growth of tumor cells. These findings suggest that overexpression of ASCT2 can promote the absorption of large amounts of glutamine and provide favorable conditions for the survival of tumor cells. However, the role of ASCT2 in the growth and development of PC and the related mechanisms have not been clarified. Recent research shows the clinicopathological significance of ASCT2 expression and proved that it is a promising pathological marker for predicting a worse outcome in PC [14]. Therefore, further exploring the biological effects of ASCT2 and the molecular mechanisms in PC cells is very important.

Here, we first revealed the critical role of ASCT2-mediated glutamine metabolism in promoting PC development and progression, and established a new mechanism that ASCT2 induced cell apoptosis via Akt/mTOR signaling pathway.

## Materials and methods

### Cell culture

Human PC cell lines (AsPC-1 and PANC-1) and a human pancreatic duct epithelial cell line (H6c7) were purchased from Beijing Beina Chuanglian Biotechnology Institute. A pancreatic cancer cell line (BxPC-3) was purchased from Wuhan Myhalic Biotechnological Co., Ltd. BxPC-3 and AsPC-1 cells were cultured in RPMI 1640 (Invitrogen, U.S.A.) containing 10% fetal bovine serum (FBS; HyClone), and PANC-1 and H6c7 cells were maintained in Dulbecco’s modified Eagle’s medium (DMEM; Invitrogen) with 10% FBS. All cell lines were maintained in a humidified incubator containing 5% CO_2_ at 37°C.

### Gene knockdown

Two pairs of primers specific to knockdown *SLC1A5* and a pair of control primers were designed and synthesized. The shRNA sequences were annealed and inserted into PLKO.1 plasmid. The plasmids, psPAx2 and pMD2.G were transfected into HEK293T cells simultaneously, and then the supernatant containing the virus in 750 µl culture medium supplemented with 30% FBS was harvested twice. The lentiviral particles in 1.5 ml culture medium were used to infect PC cells and puromycin (Sigma) was used to select stable cell lines. The silencing efficacy of the respective shRNAs was confirmed by quantitative reverse transcriptase polymerase chain reaction (qRT-PCR) and Western blotting. The shRNA sequence is as follows: PLKO.1-ASCT2 shRNA-1:

F: 5′-CCGGGCCTGAGTTGATACAAGTGAACTCGAGTTCACTTGTATCAACTCAGGCTTTTG-3′
R: 5′-AATTCAAAAAGCCTGAGTTGATACAAGTGAACTCGAGTTCACTTGTATCAACCAGGC-3′

and PLKO.1- ASCT2 shRNA-2:

F: 5′-CCGGCTGGATTATGAGGAATGGATACTCGAGTATCCATTCCTCATAATCCAGTTTTTG-3′
R: 5′-AATTCAAAAACTGGATTATGAGGAATGGATACTCGAGTATCCATTCCTCATAATCCAG-3′.

### Cell proliferation assay

Cells were seeded in a six-well plate at a density of 10^5^ cells per well. The following day, the normal culture medium was replaced with the medium containing 2 mM l-γ-glutamyl-*p*-nitroanilide (GPNA). In addition, PC stable cell lines for silencing ASCT2 were plated in a six-well plate at a density of 2 × 10^5^ cells per well. All cells were cultured for 7 continuous days. Cell growth was monitored by measuring the number of cells on the first, third, fifth and seventh days using an automatic cell counter (Cellometer Mini, Nexcelom, U.S.A.).

### Representative metabolites analysis

Glutamine consumption and the α-ketoglutarate (α-KG) and adenosine triphosphate (ATP) contents were determined using respective assay kits obtained from Biovision (Milpitas, CA, U.S.A.). In addition, ATP contents and cytoplasmic reactive oxygen species (ROS) levels were measured after the three ASCT2-depleted pancreatic cells were cultured with the ROS scavenger N-acetyl-l-cysteine (NAC, 10 mM) (Sigma) for 12 h. The 2,7-dichlorodihydrofluorescein diacetate (DCFH-DA) assay (Beyotime, China) was used to measure the intracellular ROS levels.

### Apoptosis assay

Apoptosis was detected using the Annexin V-FITC Apoptosis Kit (Beyotime, China). BxPC-3, AsPC-1 and PANC-1 cells expressing the control vector or ASCT2 shRNA were harvested by combining floating cells in the medium and adherent cells detached by 0.25% trypsin, and the cell pellets were washed once with cold phosphate-buffered saline (PBS). The cells were resuspended in 195 μL Annexin-binding buffer, and 5 μL Annexin V-FITC and 10 μL Propidium Iodide(PI) were added before incubation at room temperature for 15 min in the dark. All cells were analyzed using FACS Calibur (BD Biosciences, U.S.A.).

### RNA extraction and real-time qPCR

Total RNA was extracted from cells with TRIzol reagent (Invitrogen) according to the manufacturer’s instructions. Reverse transcription was performed using the oligo(dT) primer first-strand cDNA synthesis kit (Fermentas, Thermo Scientific, U.S.A.). The relative level of ASCT2 gene was examined using SYBR green-based real-time qPCR normalized to actin expression and presented as percentages of the control. Gene quantification was performed using the 2^−∆∆*C*_q_^ method [15]. The primers used are shown in Supplementary Table S1.

### Western blot analysis

BxPC-3, AsPC-1 and PANC-1 cells expressing the control vector or ASCT2 shRNA were treated with or without 200 nM insulin (Sigma) for 2 h. Then, these cells, H6c7 cells or tumor tissue section specimens of xenograft tumors were lysed in RIPA buffer containing PMSF and phosphotransferase inhibitor (Beyotime, China), and 50 μg total cellular protein was used for each blot, which was separated on SDS/PAGE gels and transferred to PVDF membranes. The membranes were blocked with 5% nonfat milk for 1 h at room temperature and then incubated with primary antibodies in 4°C overnight. The antibodies used were as follows: ASCT2 (1:1000, cat. no. 8057, Cell Signaling Technology, U.S.A.); Bcl-2 (1:1000, cat. no. 4223, Cell Signaling Technology, U.S.A.); Bax (1:1000, cat. no. 2772, Cell Signaling Technology, U.S.A.); AKT (1:1000, cat. no. 9272, Cell Signaling Technology, U.S.A.) and phosphoserine (Ser^473^) AKT (1:1000, cat. no. 4060, Cell Signaling Technology, U.S.A.); S6K (1:1000, cat. no. 2708, Cell Signaling Technology, U.S.A.) and phosphoserine (Thr^389^) S6K (1:1000, cat. no. 9205, Cell Signaling Technology, U.S.A.); the translation regulator 4E-binding protein 1 (4E-BP1) (1:1000, cat. no. 9644, Cell Signaling Technology, U.S.A.) and phosphoserine (Thr^37/46^)-4E-BP1 (1:1000, cat. no. 2855, Cell Signaling Technology, U.S.A.); cleaved-caspase 3 (1:1000, cat. no. 9664, Cell Signaling Technology, U.S.A.); and β-actin (1:1000, cat. no. 8457, Cell Signaling Technology, U.S.A.). After incubation with anti-rabbit IgG, HRP-linked antibody (1:1000, cat. no. 7074, Cell Signaling Technology, U.S.A.) for 1 h at room temperature, the protein bands were detected using an ECL detection system (Pierce Biotechnology, Rockford, IL, U.S.A.). The antibodies used are shown in Supplementary Table S2.

### Xenograft tumors

Male BALB/C nude mice (6 weeks, weighing 30–35 g) were purchased from Beijing Huafukang Bioscience CO., Ltd. All animals were housed with pressure-controlled ventilation at 25°C and 40–70% humidity in a 12-h light/dark cycle with food and water available *ad libitum*. Mice were divided randomly into two groups (six mice/group). Approximately 5 × 10^6^ BxPC-3 cells (control or ASCT2 shRNA) diluted in 100 μL PBS containing 50% Matrigel (BD Biosciences) were injected subcutaneously into the right flank of each nude mouse. The tumor volumes were measured every other day for 21 days, and the tumor tissues were extracted rapidly and weighed at the time of euthanasia by breaking their cervical vertebra performed a 0.2–0.5% carbon monoxide exposure. Anesthesia was not required for the subcutaneous injection.

### Immunohistochemistry (IHC)

Tumor tissues were fixed in 10% formalin and embedded in paraffin, cut into 4-μm-thick sections. The tumor sections were incubated with antibodies against ASCT2, Ki67 (1:100, cat. no. 9027 Cell Signaling Technology, U.S.A.) and cleaved caspase-3 overnight at 4°C. The remaining steps were performed using a DAKO CSA Kit (Carpinteria, Santa Clara, CA, U.S.A.). The IHC results were analyzed and scored based on the intensity of staining and the proportion of stained positive cells in a blinded manner with an integrated camera was used to acquire images from five representative fields of each section (×200).

### Statistical analysis

The data are expressed as the mean ± SD from at least three independent experiments. A two-tailed Student’s *t* test, Mann–Whitney U-test, ANOVA and Bonferroni’s corrected *t* test were adopted for the statistical analyses. Kaplan–Meier curves were estimated and compared between groups by log-rank test. *P*<0.05 was considered statistically significant.

## Results

### ASCT2 is up-regulated in PC

**Figure 1:**
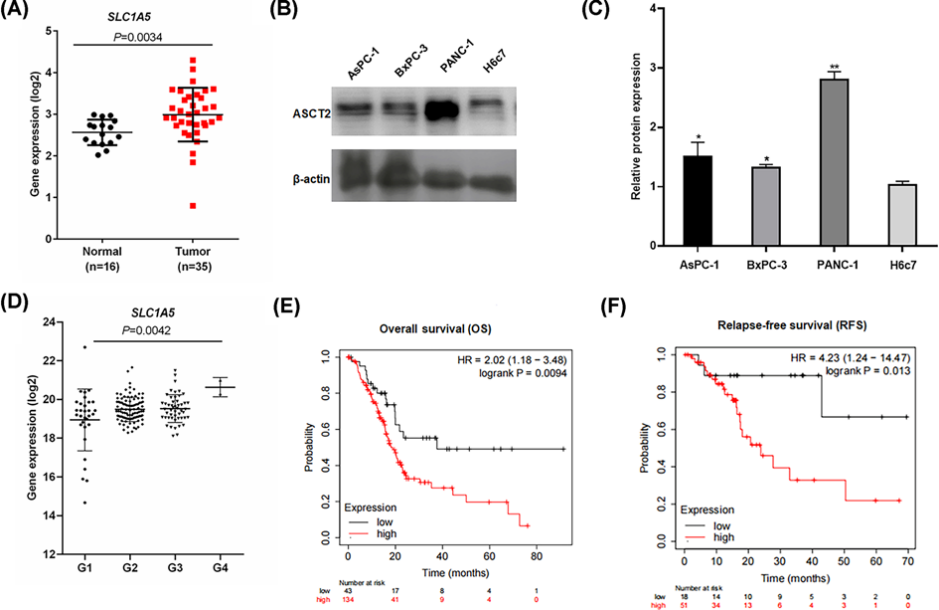
ASCT2 is up-regulated in PC (**A**) ASCT2 mRNA expression in Pei pancreas from the Oncomine database (normal *n*=16, tumor *n*=35). Mann–Whitney U-test paired *t* test: **P<0.01 versus normal pancreatic duct. (**B**,**C**) ASCT2 protein expression in PC cell lines and normal pancreatic duct cells. **P<0.01, *P<0.05 versus H6c7 cells. (**D**) ASCT2 mRNA expression among Gleason grades in the TCGA dataset. (Gleason 1, *n*=31; Gleason 2, *n*=97; Gleason 3, *n*=50; Gleason 4, *n*=2. Mann–Whitney U-test: *P*=0.0042 versus Gleason 1). (**E**,**F**) The Kaplan–Meier plotter downloaded from the Kaplan–Meier plotter database (PC), the Kaplan–Meier plotter of OS ((E), HR = 2.02, *P*=0.0094, log-rank test) and relapse-free survival ((F), HR = 4.23, *P*=0.013, log-rank test).

To investigate the expression levels of ASCT2, we analyzed the mRNA levels of ASCT2 in PC and normal pancreatic tissues using Oncomine microarray gene expression datasets (www.oncomine.org). The *SLC1A5* mRNA levels were up-regulated in PC tissues compared with normal pancreatic duct tissues according to the Pei pancreas dataset in Oncomine (Figure 1A, *P*=0.0034, Mann–Whitney U-test). The analysis of ASCT2 protein showed that ASCT2 was expressed at different levels in PC cell lines and normal pancreatic duct cells and highly expressed in BxPC-3, PANC-1 and AsPC-1 cells, with lower levels detected in H6c7 cells (^*^P<0.05, ^**^P<0.01, Figure 1B,C). *SLC1A5* expression among tumors of different Gleason grades in the The Cancer Genome Atlas (TCGA) dataset was analyzed, and the results showed that *SLC1A5* expression increased as the stage evolved (Figure 1D, *P*=0.0042, Bonferroni’s corrected *t* test). Additionally, we also used the public cancer database KM plotter to analyze the survival of PC patients with different expressions of *SLC1A5.* As shown in Figure 1E,F, *SLC1A5* predicted as a negative index for PC patients. In specific, PC patients with high *SLC1A5* expression had a shorter overall survival (OS) (Figure 1E, Hazard ratio (HR)= 2.02, *P*=0.0094, log-rank test) or relapse-free survival (Figure 1F, HR = 4.23, *P*=0.013, log-rank test) than patients with low expression. The results above indicated that *SLC1A5* was functional in PC.

### ASCT2 knockdown suppresses glutamine metabolism in PC

**Figure 2:**
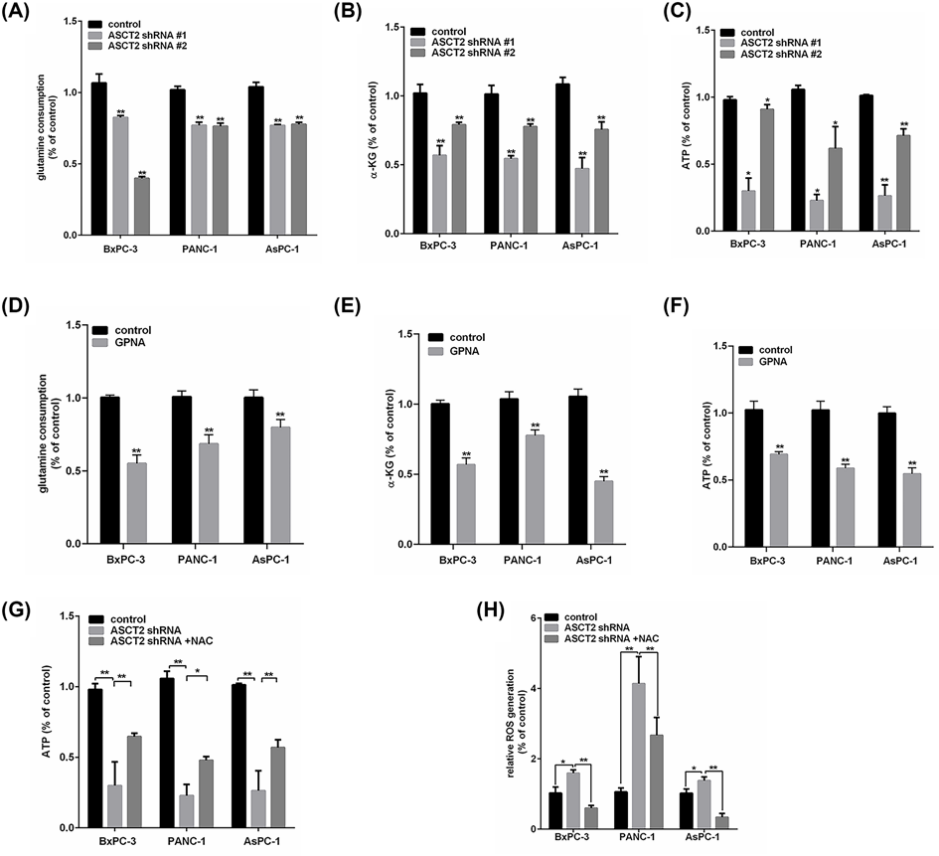
Effect of ASCT2 knockdown or chemical inhibition on cell glutamine metabolism in BxPC-3, PANC-1 and AsPC-1 cells (**A–C**) Relative changes in (A) glutamine consumption, (B) α-KG production and (C) intracellular ATP content in BxPC-3, PANC-1 and AsPC-1 cells upon ASCT2 knockdown (*P<0.05, **P<0.01, versus the control group). (**D–F**) Relative changes in (D) glutamine consumption, (E) α-KG production and (F) intracellular ATP content in BxPC-3, PANC-1 and AsPC-1 cells in the presence of the ASCT2 inhibitor GPNA (2 mM) (*P<0.05, **P<0.01, versus the control group). (**G,H**) Relative changes in (G) intracellular ATP content, (H) ROS generation for control or shASCT2 in the presence or absence of NAC in BxPC-3, PANC-1 and AsPC-1 cells (*P<0.05, **P<0.01, versus the control group or shASCT2).

**Figure 3:**
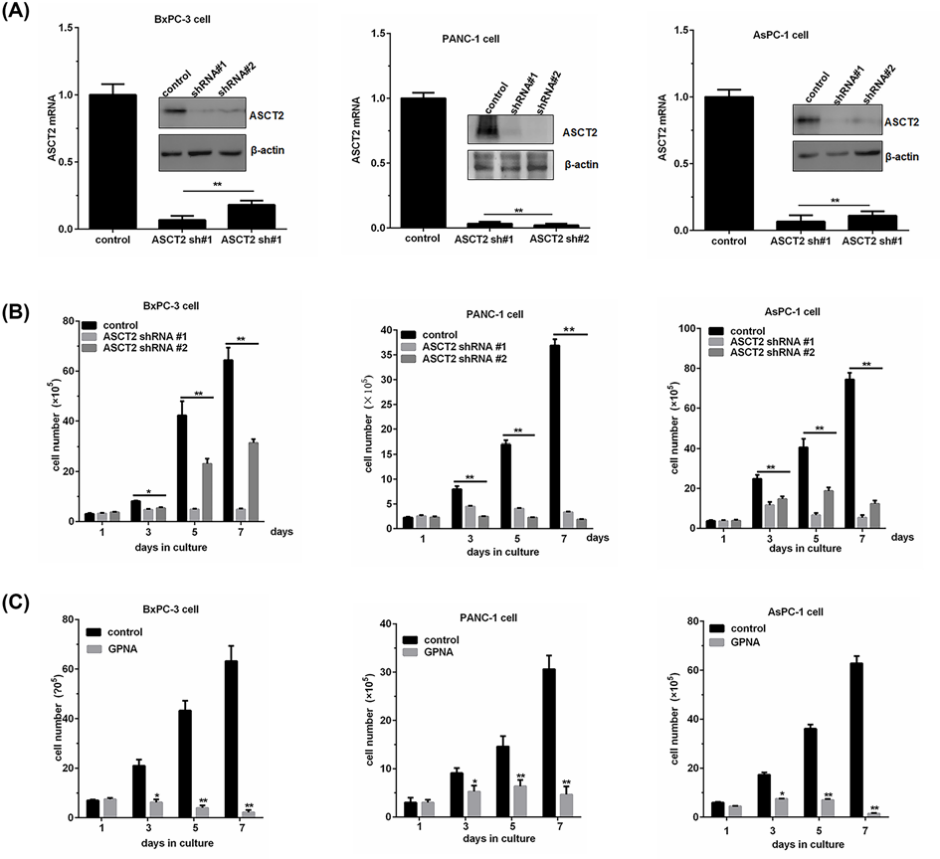
Effect of ASCT2 knockdown or chemical inhibition on cell proliferation in PC cells (**A**) Depletion of ASCT2 expression by two specific shRNAs in BxPC-3, PANC-1 and AsPC-1 cells. The relative ASCT2 mRNA levels were quantified by real-time qPCR; the data shown are an average of triplicates from a single cDNA. The ASCT2 protein levels were analyzed by Western blotting, and β-actin was used as a loading control. (**B**) The proliferation of BxPC-3, PANC-1 and AsPC-1 cells over 7 days as measured by serial cell counts after ASCT2 knockdown. (**C**) The cell growth of BxPC-3, PANC-1 and AsPC-1 cells was examined in the presence of the ASCT2 inhibitor GPNA (2 mM) for 1, 3, 5, and 7 days. The data are shown as an average of triplicates. *P<0.05, **P<0.01, versus the control group.

To examine whether ASCT2-mediated glutamine uptake sustains tricarboxylic acid (TCA) cycle anaplerosis and ATP production, we depleted ASCT2 expression by two specific shRNAs in three PC cell lines first and then measured glutamine consumption, α-KG production (a TCA cycle intermediate) and ATP generation. Our results showed that the knockdown of endogenous ASCT2 was >80% efficient (^**^P<0.01), as determined by real-time qPCR and the immunoblot analysis (Figure 3A).In addition, we observed that glutamine consumption, α-KG production and ATP generation decreased to different degrees in the ASCT2-depleted PC cells with (GPNA, 2 mM) treatment compared with those with the control treatment (^*^P<0.05, ^**^P<0.01) (Figure 2A–F). To further investigate the effect of ASCT2 on redox homeostasis in the cell, we used the fluorescent dye DCFH-DA to measure intracellular ROS production. As shown in Figure 2H, ASCT2 elimination caused a dramatic increase in ROS production compared with that in the control (^*^P<0.05, ^**^P<0.01). However, when the three ASCT2-depleted PC cell lines were cultured with NAC for 12 h, the ATP content increased, but ROS production declined significantly (^*^P<0.05, ^**^P<0.01) (Figure 2G,H). The results suggest that NAC might significantly reverse the effect of ASCT2 knockdown on ATP generation and oxidative stress.

### ASCT2 knockdown inhibits the growth and induces programmed cell death (apoptosis) of PC cells

**Figure 4:**
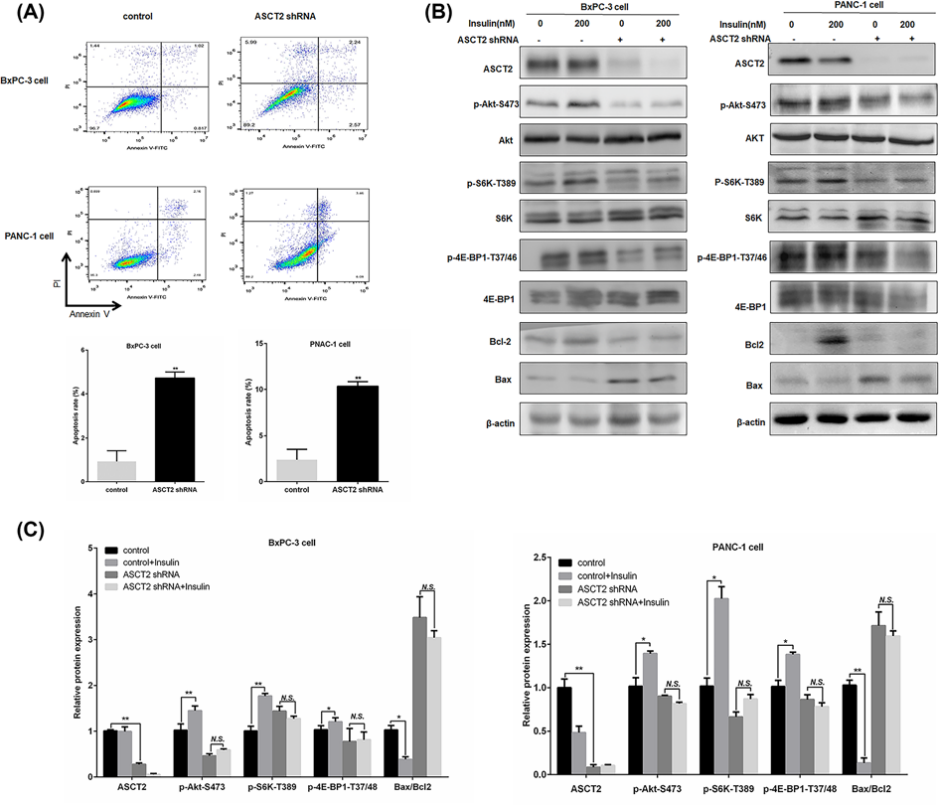
Effect of ASCT2 knockdown on cell apoptosis through the Akt/mTOR signaling pathway (**A**) The rate of cell apoptosis was detected through flow cytometry in BxPC-3 and PANC-1 cells upon ASCT2 knockdown. (**B,C**) BxPC-3 and PANC-1 cells were infected with a control or ASCT2 lentiviral shRNA and then treated with or without 200 nM insulin for 2 h. A Western blotting analysis of the expressions of AKT, p-AKT (Ser^473^), 4E-BP1, p-4E-BP1, Bcl-2, and Bax proteins. β-actin was used as the loading control. *P<0.05, **P<0.01, versus the control group.

To directly evaluate ASCT2 function in cell growth, we quantified viable cells cultured in ASCT2-depleted cells using cell number counts. Whereas the control cells proliferated substantially over time, the ASCT2-depleted cells showed a pronounced reduction in growth (^*^P<0.05, ^**^P<0.01) (Figure 3B). We also assessed whether the ASCT2 inhibitor GPNA treatment altered PC cell growth. As shown in (Figure 3C), after 7 days of treatment with 2 mM GPNA, cell growth was significantly decreased compared with that in the control in BxPC-3, PANC-1 and AsPC-1 cells (^*^P<0.05, ^**^P<0.01). These results suggested that ASCT2 inhibition suppressed the proliferation of PC cells. To examine the effect of ASCT2 knockdown on apoptosis in PC, we further performed a flow cytometry assay to measure the apoptosis ratio in three PC cell lines with ASCT2 knockdown. Compared with the control group, the total apoptosis ratio was significantly increased after ASCT2 knockdown (from 0.93 to 4.72% in BxPC-3 cells, from 2.39 to 10.3% in PANC-1 cells, and from 2.46 to 10.95% in AsPC-1 cells; (Figure 4A), Supplementary Figure S1A) (all ^*^P<0.01). Altogether, these results suggest that ASCT2 might play an important role in the growth and survival of PC cells.

### ASCT2 knockdown induces apoptosis via the Akt/mTOR pathway in PC

To determine whether ASCT2 knockdown induced apoptosis we further analyzed the expression levels of the apoptosis-related proteins. As shown in Supplementary Figure S1B, C, cleaved caspase-3 protein expression levels increased 20.3 ± 0.02%, 76.6 ± 0.03% and 67.4 ± 0.02% and the ratio of Bax/Bcl-2 was also increased 1.2-, 1.2- and 2.6-fold, respectively, in BxPC-3, PANC-1, and AsPC-1 cells expressing shASCT2, compared with those expressing the nontargeting control shRNA (*n*=3, ^**^P<0.01). It is reported that insulin has anti-apoptotic activity through activation of the PI3K/Akt pathway [16]. To further verify whether ASCT2 knockdown induced apoptosis by the Akt/mTOR pathway, we treated ASCT2-knockdown cells with insulin for 2 h. As shown in (Figure 4B,C), insulin increased the expression levels of phosphorylated AKT and mTOR downstream factors, i.e., S6K and 4E-BP1 by approximately 41.8%, 75.9%, 16.7% in BxPC-3 cells, and by approximately 37%, 98.8%, 36.5% in PANC-1 cells, respectively. In addition, insulin decreased the ratio of Bax/Bcl-2 by approximately 62.5% in BxPC-3, by approximately 87% in PANC-1 cells (*n*=3, ^*^P<0.05, ^**^P<0.01). However, there is no significant difference between with and without treatment after ASCT2 knockdown (*n*=3, all *P*>0.05). These results indicate that ASCT2 knockdown might induce apoptosis in PC cells by affecting the expression of Bcl-2 and Bax and inhibiting the Akt/mTOR signaling pathway.

### ASCT2 knockdown attenuates tumor growth in nude mice bearing BxPC-3 xenograft tumors *in vivo*

**Figure 5:**
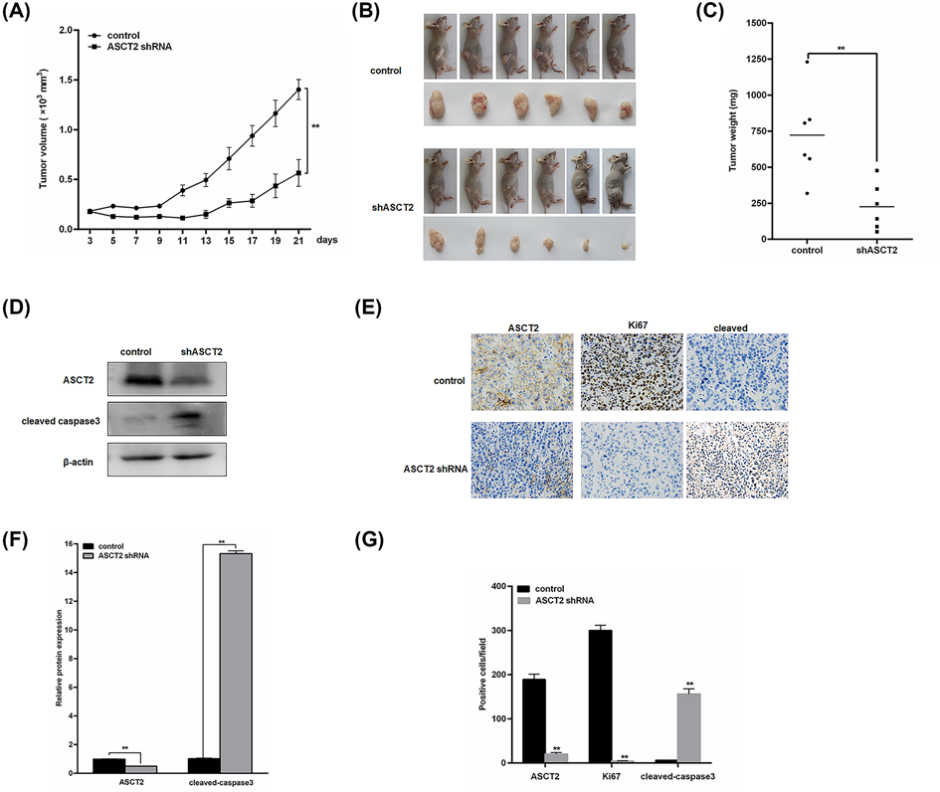
ASCT2 knockdown attenuates tumor growth in nude mice bearing BxPC-3 xenograft tumors *in vivo* (**A**) The xenograft tumors of BxPC-3 cells (shRNA-Control, *n*=6; shRNA-ASCT2, *n*=6) were measured every 2 days to assess their growth. Two perpendicular diameters of the xenograft tumor were examined and converted into a tumor volume (mm^3^) using the formula (a × b^2^)/2, where ‘a’ and ‘b’ refer to the longer and shorter dimensions, respectively. **P<0.01 versus the control group. (**B**,** C**) The tumors were removed and photographed after the mice were anesthetized. The final tumor weights in each group were measured at the end of the experiment. Scale bar, 1 cm. (**D**, **F**) The Western blot analysis of the protein expression of ASCT2 and cleaved caspase-3 in tissue sections of xenografts treated with either ASCT2-shRNA or nontargeting shRNA. β-Actin was used as the loading control. (**E**) Representative staining of ASCT2, Ki67 and cleaved caspase-3 in tumor sections derived from (B). Magnification = 200×. (**G**) Assessment of ASCT2, Ki67 and cleaved caspase-3 expression using the semiquantitative scoring system. **P<0.01 versus the control group.

To assess the ability of ASCT2 ablation to inhibit the tumorigenic capacity of PC, we generated BxPC-3 cells expressing a control or ASCT2-specific shRNA. Then, BxPC-3 cells expressing a control or ASCT2-specific shRNA were subcutaneously injected into the right flank of nude mice. Tumor size was analyzed every other day for 21 days, showing a significant decrease in shASCT2 tumor size compared with their control shRNA counterparts (^**^P<0.01) (Figure 5A). Mice were euthanized after 21 days, and the tumors were isolated, removed, imaged and weighed. Strikingly, compared with the control shRNA tumors, the shASCT2 tumors showed significant reductions in both size and weight (all ^**^P<0.01) (Figure 5B,C). Western blots of the xenograft tumors revealed that ASCT2 was decreased in shASCT2 tumors compared with the control tumors, while the apoptosis biomarker cleaved caspase-3 showed elevated levels (all ^**^P<0.01) (Figure 5D,F). IHC analysis revealed that the protein expression levels of ASCT2 and Ki67 were lower in shASCT2 tumors than in control tumors, while the level of cleaved caspase-3 was significantly increased (all ^**^P<0.01) (Figure 5E,G). Consistent with the results obtained *in vitro* (Figure 3), the depletion of ASCT2 expression profoundly inhibited tumor cell proliferation, concomitant with a dramatic increase in cell death *in vivo*. Taken together, our results demonstrate that targeting ASCT2 may be an effective approach to PC therapy.

## Discussion

The high-expression levels of SLC1A5 have been reported to be overexpressed in many human cancers [10–13,17,18]. Our results also show that ASCT2 mRNA is significantly overexpressed in PC tissue samples or PC cells compared with normal pancreatic ductal cells. Considerable evidence support that increased ASCT2 expression is closely related to poor prognosis, such as non-small cell lung cancer [10], colorectal cancer [9], prostate cancer [19] and gastric cancer [20]. Our study also demonstrated that higher expression of ASCT2 in PC was significantly correlated with TNM stages and poor prognosis of these individuals through the Kaplan–Meier plotter analysis. Therefore, ASCT2 could be considered as a promising pathological marker for predicting a worse outcome in PC, which was in accordance with previous literature [14].

The ASCT2 transporter has been recently reported to be responsible for glutamine uptake in PC cells [21]. but little is known about how ASCT2 is involved in glutamine metabolism in PC cells. Our results suggest that blocking ASCT2 using either chemical or genetic means (ASCT2 shRNA knockdown) significantly decreased glutamine consumption, α-KG production and ATP generation. These results indicate that ASCT2-mediated glutamine uptake sustains TCA cycle anaplerosis and ATP production.

Son et al. reported that glutamine deprivation leads to an increase in ROS in human pancreatic ductal adenocarcinoma (PDAC) cells [22]. Newsholme et al. released research suggesting that glutamine is a precursor of reduced glutathione (GSH) and can also prevent the mitochondria from oxidative damage [23]. Tamoxifen and Raloxifene can block the cellular uptake of glutamine by inhibiting ASCT2, thus reducing the intracellular GSH level and producing certain cytotoxicity. Concurrently, the cytotoxicity of the two drugs can be reversed by increasing GSH through the addition of NAC and 17-β-estradiol treatment [24]. Therefore, we speculate that ASCT2-mediated glutamine uptake could play a major role in maintaining redox balance. To test this hypothesis, we assessed ROS levels and ATP production upon ASCT2 knockdown in the absence or presence of NAC. Indeed, ASCT2 knockdown induces ROS and reduces ATP generation, and NAC partially attenuates the increase in ROS and reduction in ATP generation. Together, these data support the idea that ASCT2 is used by PC cells to regulate glutamine metabolism and maintain redox homeostasis.

There were many reports showed that ASCT2 performed a significant function in cancer cell proliferation and apoptosis [9,13,25,26]. However, there is no convincing data to implicate ASCT2 involved in the initiation and progression of PC. Our study showed that blockade of ASCT2-mediated glutamine regulation by GPNA or depletion of ASCT2 expression by specific shRNA inhibited the growth of PC cells in culture. In addition, knockdown of ASCT2 induced apoptotic cell death. Furthermore, we elucidated the mechanisms by which ASCT2 knockdown induced cell apoptosis. ASCT2 suppression significantly decreased Bcl-2 expression and increased Bax and cleaved caspase-3 expression. Therefore, the mechanisms of apoptosis were related to the mitochondrial pathway (Bcl-2/Bax).

Furthermore, we confirmed our findings *in vivo* using a model of human PC xenografts in nude mice. We found that knocking down ASCT2 inhibited the relative tumor volume and weight. According to the IHC and Western blotting results, the expression levels of ASCT2 and Ki67 protein were decreased while the cleaved caspase-3 expression levels were clearly increased in shASCT2 tumors compared with control tumors. These findings reveal that suppressing ASCT2 inhibited the formation of subcutaneous tumor nodules by inhibiting cell proliferation and promoting apoptosis. Combined with the results of the *in vitro* experiment, these data suggest that ASCT2 could be a potential candidate therapeutic target in PC.

It is reported that the mTOR inhibitor, rapamycin decreased ASCT2 mRNA expression in SK-Hep1 cells, that in turn depressed the mTOR signaling by decreasing the p70 S6K1 and eukaryotic initiation factor 4E-BP1 phosphorylation in a reciprocal effect [27]. ASCT2 suppression significantly decreased glutamine uptake, which led to a decrease in the phosphorylation of Akt kinase on Ser^473^, the 70-kDa ribosomal protein S6 kinase and eukaryotic initiation factor-4E (eIF4E) binding protein-1 (4E-BP1) in prostate cancer [13], breast cancer [28] and hepatocellular carcinoma [27]. However, very recently, Bothwell’s group reported the link between ASCT2 expression and mTOR function initially, proposed that target suppression or knockout of ASCT2 is unaffected cellular proliferation and mTORC1 signaling in either liver cancer cell type [29]. What is ASCT2 expression and the mTOR signaling pathway in PC? Our study showed that the phosphorylation of Akt, S6K and 4E-BP1 induced by insulin was notably attenuated after ASCT2 knockdown, which indicate that ASCT2 knockdown induces cell apoptosis through the Akt/mTOR signaling pathway.

In conclusion, we have revealed connections between ASCT2 and the development and progression of PC *in* *vivo* and *in* *vitro*. ASCT2 is highly expressed in PC, and the depletion of ASCT2 expression likely inhibits glutamine metabolism, ROS production and PC cell growth and survival. Our results also indicate a potential mechanism for apoptosis caused by ASCT2 knockdown. Therefore, ASCT2 may be a promising target for new drugs to treat PC.

## Supplementary Material

Supplementary Figure S1 and Tables S1-S2

## Data Availability

All supporting data are included in supplementary files. Raw data associated with the paper are available and can be accessed by contacting the authors.

## Abbreviations

α-KG: α-ketoglutarate
ASCT2/SLC1A5: alanine-serine-cysteine transporter 2
ATP: adenosine triphosphate
DCFH-DA: dichlorodihydrofluorescein diacetate
DMEM: Dulbecco’s modified Eagle’s medium
eIF4E: eukaryotic initiation factor-4E
FBS: fetal bovine serum
GPNA: l-γ-glutamyl-*p*-nitroanilide
GSH: glutathione
HR: Hazard Rate
IHC: Immunohistochemistry
NAC: N-acetyl-l-cysteine
OS: overall survival
PBS: phosphate-buffered saline
PC: Pancreatic cancer
PDAC: pancreatic ductal adenocarcinoma
PI: Propidium Iodide
qRT-PCR: quantitative reverse transcriptase polymerase chain reaction
ROS: reactive oxygen species
TCA: tricarboxylic acid
TCGA: The Cancer Genome Atlas
4E-BP1: translation regulator 4E binding protein 1
