# Supplementary material for: Targeting ASCT2-mediated glutamine metabolism inhibits proliferation and promotes apoptosis of pancreatic cancer cells
Source: Biosci Rep. 2022 Mar 16;42(3):BSR20212171. doi: 10.1042/BSR20212171 (PMC8935385; doi:10.1042/BSR20212171)
Supplement: Supplementary Figure S1 and Tables S1-S2 [file BSR-2021-2171_supp.pdf]

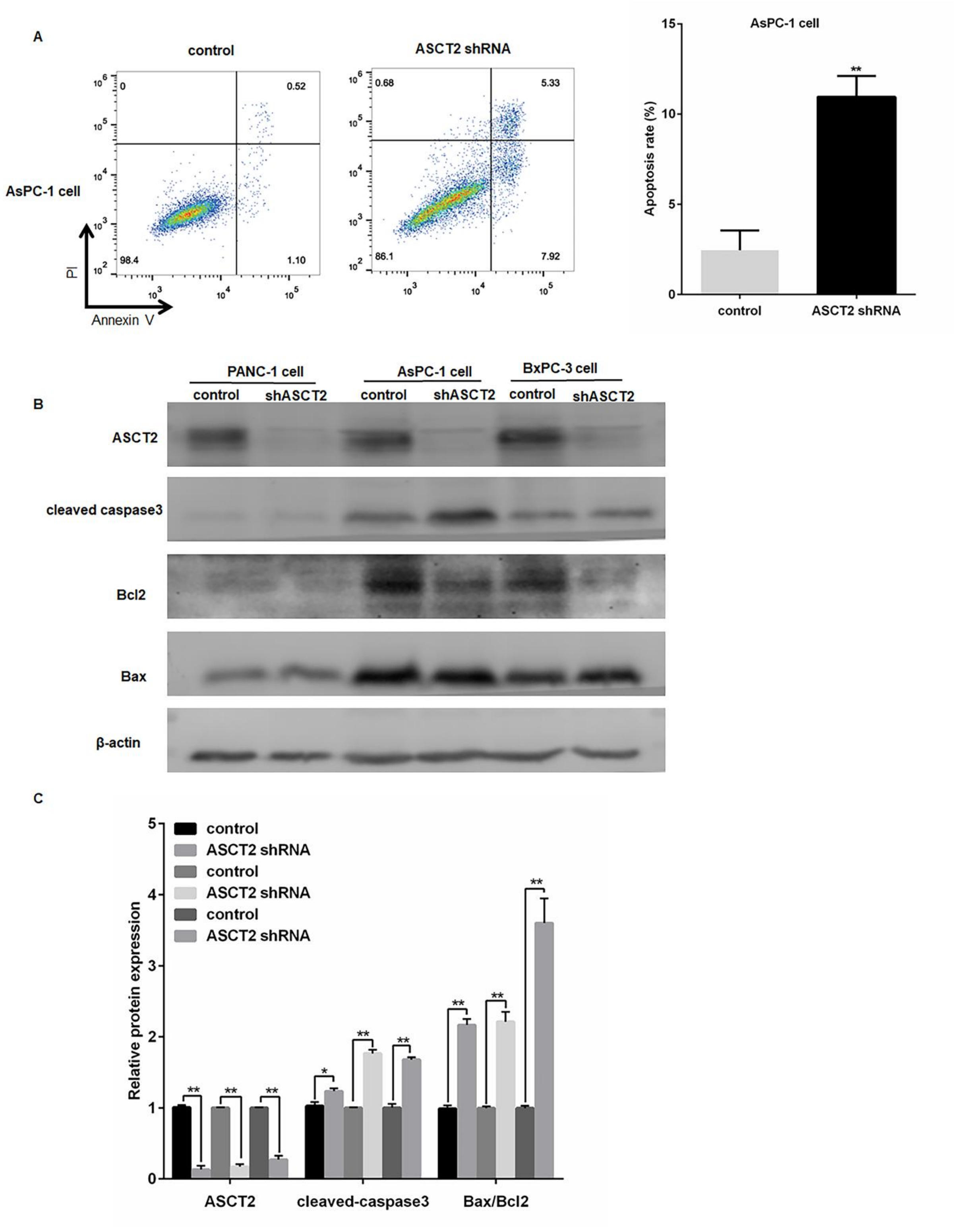

Figure S1. Effect of ASCT2 knockdown on cell apoptosis. (A) The rate of cell apoptosis was detected through flow cytometry in AsPC1 cells upon ASCT2 knockdown. (B-C) BxPC3, PANC-1 and AsPC1 cells were respectively infected with a control or ASCT2 lentiviral shRNA. A Western blot analysis of the expressions of Bcl-2, Bax and cleavage-caspase 3 proteins. β-actin was used as the loading control. $^{*}P$ <0.05, $^{**}P$ <0.01, versus the control group.

Supplementary Table 1 Primer sequences used in the study.

primer name	sequence (5' to 3')
qPCR primers	
ASCT2 F	GCAGTCCTTGGACTTCGTAAA
ASCT2 R	ATGATGGCCAGAGTGAGGAC
β-actin F	AGCCTCGCCTTTGCCGA
β-actin R	GCGCGGCGATATCATCATC

Supplementary Table 2   Antibody used in the study.

Name	Cat.No.	antibody dilutions	Source/Isotype	MW (kDa)
ASCT2 (D7C12) Rabbit mAb	8057	1:1000	Rabbit	49, 75
Bcl-2 (D55G8) Rabbit mAb	4223	1:1000	Rabbit	26
Bax	2772	1:1000	Rabbit	20
Akt	9272	1:1000	Rabbit	60
Phospho-Akt (Ser473) (D9E) XP® Rabbit mAb	4060	1:1000	Rabbit	60
p70 S6 Kinase (49D7) Rabbit mAb	2708	1:1000	Rabbit	70, 85
Phospho-p70 S6 Kinase (Thr389) Antibody	9205	1:1000	Rabbit	70, 85
4E-BP1 (53H11) Rabbit mAb	9644	1:1000	Rabbit	15-20
Phospho-4E-BP1 (Thr37/46) (236B4) Rabbit mAb	2855	1:1000	Rabbit	15-20
Cleaved Caspase-3 (Asp175) (5A1E) Rabbit mAb	9664	1:1000	Rabbit	17, 19
β-Actin (D6A8) Rabbit mAb	8457	1:1000	Rabbit	45
Anti-rabbit IgG, HRP-linked Antibody	7074	1:1000	Goat	
